# Spillover Trends of Child Labor During the Coronavirus Crisis- an Unnoticed Wake-Up Call

**DOI:** 10.3389/fpubh.2020.00488

**Published:** 2020-09-04

**Authors:** Md. Abdul Ahad, Yvonne K. Parry, Eileen Willis

**Affiliations:** ^1^Department of Rural Sociology & Development, Sylhet Agricultural University, Sylhet, Bangladesh; ^2^College of Nursing and Health Sciences, Flinders University, Adelaide, SA, Australia

**Keywords:** child labor, Coronavirus, unemployment, poverty, school

## Introduction

The worldwide surge of Covid-19 pandemic threatens the lives of impoverished and vulnerable segments of society. Children, particularly, are at greater risk of maltreatment and neglect rather than disease contamination. Children in labor are the highly overlooked topic in research and national plans than other aspects of child abuse provoked by the Covid-19 outbreak.

## Discussion

According to estimates of the International Labour Organization (ILO) (1), there is still staggering 152 million child laborers, of which around 73 million are in hazardous work. This estimate of child laborers was around 210 million in 2000. Between 2002 and 2006, an 11% fall of child labor was noticed globally with a sharp decline of hazardous child labor (2). The present prevalence of child labor is deemed to be slowing in comparison with previous decades because of increased education, booming economies and improved labor laws (3). This has resulted in a significant proportion of child laborers being able to attend school. Unfortunately, it is predicted that the effects of the on-going Covid-19 pandemic and public health measures will break this cycle and revitalizing the presence of children in this illegitimate activity (4).

Public health measures such as lockdowns or social isolation during the Covid-19 outbreak have resulted in fears of economic recession and falls in stock market indices in most countries. Border shutdowns and travel restriction have subsequently led to a crash in demand for consumer goods, disruption in supply chains, falling product prices, low investment in manufacture, and curbed remittances between countries (4, 5). These unprecedented disruptions in the global production market have increased the risk of unemployment evoked by the Covid-19 outbreak. The ILO (6) reported that the dramatic decline of jobs and aggregated hours of work have already affected the world's 3.3 billion workers during the recent lockdown measures. The United Nations (UN) labor body projected that the economic disruption caused by the Covid-19 outbreak may wipe out 195 million jobs worldwide (7). In the USA alone, around 30.3 million initial claims for unemployment insurance were lodged between March 21 to April 25 of 2020 (8). This forecasts a massive increase in job losses during this outbreak both in high- and middle-income countries.

Despite the comprehensive surveillance strategies, many low- and middle-income countries (LMICs) are overwhelmed by the precipitous spread of Covid-19 contamination. Bong et al. (9) postulate that LMICs will suffer from overcrowded hospitals as a result of Covid-19 infected patients due to large populations, disregarding the imposed public health measures, and insufficient health care facilities. The aftermath may not be limited to respiratory infections but may result in precarious social and economic vulnerability in many afflicted and impoverished countries. The United Nations Development Programme (UNDP) report stresses that the forecast of income opportunity loss in poor or developing countries could exceed US $220 billion, which could take many years to recover (10). This economic recession would further expose rapid escalation of unemployment and poverty in these countries. The ILO and United Nations Children's Fund (UNICEF) (4) estimate that around 40 to 60 million people could fall into extreme poverty this year alone. The effects could also result in prolonged socio-psychological stressors.

A significant proportion of poor communities in the LMICs will experience extreme exploitation and hunger, as many parents fall into a poverty cycle. For instance, around 9.1% of the population in Sub-Saharan Africa are already in extreme poverty including 3.9 million children who are deprived of food at the end of first 8-weeks of the lockdown during the Covid-19 outbreak (11). Sufficient financial supports from public or private stakeholders to these impoverished people of many LMICs is also not evident in the media or in current research. The pressure of poverty will force parents to seek employment prospects for their children. Becker (12) suggests that one of the effects of Covid-19 may result in parental unemployment, sickness or death, a drop in household incomes, children will be forced into labor to meet the basic needs of their family members. The escalating prospect of child labor is associated with the upsurge of poverty rates. Sasmal and Guilen (13) note that when poverty persists, parents are forced to send their children to work.

The World Economic Forum reports that in the Ivory Coast, there has been a 10% decrease in households' income which led to an increase in child labor of 5% (14). Children from marginalized minority groups, homeless, migrant refugees, disabled, and those living in war or disaster-prone areas are at particular risk of extreme deprivation or starvation in this unprecedented crisis. The ILO and UNICEF (4) estimates that a 1% rise in poverty can result in a 0.7% surge in child labor. However, child labor violates the fundamental rights of children including their education, health, recreation, and other development prospects. The engagement in hazardous job in exhausting environment and working long hours will be prompted by poverty.

School closures during the Covid-19 outbreak will also trigger a resurgence of child labor plight. Research reveals that children who do not attend school are more likely to join the workforce than those who go to school (12). Past experiences reveal that in every pandemic, this complex experience emerges. For example, school closures during the Ebola pandemic crisis of 2014 resulted in an escalation of child labor (15). Of note, children lacking access to technology and the internet, will be unable to join in the online remote learning during the school closures. Kluttz (16) suggests that child labor is not simply an indicator of poverty, it is also a consequence of poor access to education. Even when schools re-open, many parents or guardian may no longer be able to afford to continue to pay for their child's schooling. Rural out-of-school children could be more vulnerable than urban child laborers. Due to poor maintenances of lockdown policies in rural precincts, the shutdown of schools and poor access to online education, families may tend to propel their children into work particularly in the rural agriculture sectors. Roughly 71% of child laborers are involved in the agriculture sector doing hazardous works. A significant proportion of them are bonded laborers (1). The exploitation of children in labor due to school closure would particularly be acute in Africa and Asia, where the majority of child laborers work in agriculture.

These explicit risk factors of the Covid-19 pandemic may apparently accelerate the growth of child labor around the globe. This sudden rise in the prevalence of child labor may wipe out the target of achieving Sustainable Development Goal 8.7, which focus on ending child labor in all its form such as eradicating forced labor, modern slavery, human trafficking and elimination of the worst forms of child labor, including recruitment and use of child soldiers by the year 2025 through integrated thinking, co-ordinated actions, effective policy practices, and use of resources (1). Therefore, to ensure the current gains are not lost, the governments of the affected countries should take immediate measures. Alongside raising public awareness to prevent child labor in this unprecedented crisis, the respective governments must set up cash transfer programmes with a priori-focus on the impoverished and vulnerable households and promote online education platforms with the necessary aids for vulnerable children.

## Author Contributions

MA, YP, and EW wrote this opinion paper together. MA developed the idea and wrote the first draft of the opinion paper. All authors contributed to the article and approved the submitted version.

## Conflict of Interest

The authors declare that the research was conducted in the absence of any commercial or financial relationships that could be construed as a potential conflict of interest.

## References

[1] International Labor Organization Global Estimates of Child Labour: Results and Trends, 2012-2016. International Labor Office, Geneva (2017). Available online at: https://www.ilo.org/wcmsp5/groups/public/—dgreports/—dcomm/documents/publication/wcms_575499.pdf

[2] International Labour Organization The End of Child Labour: Within Reach. Global Report Under the follow-Up to the ILO Declaration on Fundamental Principles and Rights at Work. International Labour Office, Geneva (2006). Available online at: https://www.ilo.org/global/publications/ilo-bookstore/order-online/books/WCMS_PUBL_9221166031_EN/lang–en/index.htm (accessed May 24, 2020).

[3] Radfar A Asgharzadeh S Quesada F Filip I. Challenges and perspectives of child labor Industrial Psychiatry J. (2018) 27:17–20. 10.4103/ipj.ipj10514 30416287 PMC6198592

[4] International Labour Organization and United Nations Children's Fund. ‘COVID-19 and Child Labour: A Time of Crisis, A Time to Act’. New York (2020). Available online at: https://www.ilo.org/wcmsp5/groups/public/—ed_norm/—ipec/documents/publication/wcms_747421.pdf (accessed June 4, 2020).

[5] Nicola M Alsafi Z Sohrabi C Kerwan A Al-Jabir A Iosifidis C. The socio-economic implications of the coronavirus pandemic (COVID-19): a review. Int J Surg. (2020) 78:185–93. 10.1016/j.ijsu.2020.04.018 32305533 PMC7162753

[6] International Labor Organization ILO Monitor: COVID-19 and the World of Work. 2nd ed. Updated Estimates and Analysis. International Labor Office (2020). Available online at: https://www.ilo.org/wcmsp5/groups/public/—dgreports/—dcomm/documents/briefingnote/wcms_740877.pdf (accessed June 3, 2020).

[7] United Nations Covid-19 to Wipe Out Equivalent of 195m Jobs, Says UN Agency. The Guardian (2020). Available online at: https://www.theguardian.com/world/2020/apr/07/covid-19-expected-to-to-wipe-out-67-of-worlds-working-hours (accessed June 15, 2020).

[8] Sahin A Tasci M Yan F The Unemployment Cost of COVID-19: How High and How Long?. Federal Reserve Bank of Cleveland (2020).

[9] Bong CL Brasher C Chikumba E McDougall R Mellin-Olsen J Enright A. The COVID-19 pandemic: effects on low and middle-income countries. Anesth Analg. (2020) 131:86–92. 10.1213/ANE.0000000000004846 32243287 PMC7173081

[10] Suleymanove R Developing Countries Face Economic Collapse in COVID-19 Fight: UN, ALJAZEERA, Qatar (2020). Available online at: https://www.aljazeera.com/ajimpact/developing-countries-face-economic-collapse-covid-19-fight-200330003332689.html (accessed June 12, 2020).

[11] Teachout M Zipfel C The Economic Impact of COVID-19 Lockdowns in Sub-Saharan Africa. International Growth Centre (2020). Available online at: https://www.theigc.org/publication/lockdowns-in-africa/ (accessed June 8, 2020).

[12] Becker J Covid-19 Pandemic Threatens Progress on Child Labor. Human Rights Watch. (2020). Available online at: https://reliefweb.int/report/world/covid-19-pandemic-threatens-progress-child-labor (accessed June 3, 2020).

[13] Sasmal J Guillen J Poverty, educational failure and the child-labour trap: the Indian experience. Glob Bus Rev. (2015) 16:270–80. 10.1177/0972150914564419

[14] Moore K COVID-19 Heightens the Risk of Child Labour. This is How We Can Tackle It. World Economic Forum (2020). Available online at: https://www.weforum.org/agenda/2020/05/covid-19-heightens-the-risk-of-child-labor-but-there-is-a-path-to-child-labor-free-cocoa/ (accessed June 8, 2020).

[15] International Labor Organization COVID-19 Impact on Child Labour and Forced Labour: The Response of the IPEC+ Flagship Programme. International Labour Office, Geneva (2020). Available online at:https://www.ilo.org/wcmsp5/groups/public/—ed_norm/—ipec/documents/publication/wcms_745287.pdf

[16] Kluttz J Re-evaluating the relationship between education and child labour using the capabilities approach: policy and implications for inequality in Cambodia. Theory Res in Educ. (2015) 13:165–79. 10.1177/1477878515593886

